# Deleterious effects of lymphocytes at the early stage of neurodegeneration in an animal model of amyotrophic lateral sclerosis

**DOI:** 10.1186/1742-2094-8-19

**Published:** 2011-02-23

**Authors:** Satoru Tada, Tatsusada Okuno, Teruhito Yasui, Yuji Nakatsuji, Tomoyuki Sugimoto, Hitoshi Kikutani, Saburo Sakoda

**Affiliations:** 1 Department of Neurology, Osaka University Graduate School of Medicine, 2-2 Yamadaoka, Suita, Osaka, Japan; 2 Department of Molecular Immunology, Research Institute for Microbial Disease, Osaka University, 3-1 Yamadaoka, Suita, Osaka, Japan; 3 Department of Immunopathology, Research Institute for Microbial Disease, Osaka University, 3-1 Yamadaoka, Suita, Osaka, Japan; 4 Department of Bio-medical statistics, Osaka University Graduate School of Medicine, 2-2 Yamadaoka, Suita, Osaka, Japan; 5 National Toneyama Hospital, 5-1-1 Toneyama, Toyonaka, Osaka, Japan

## Abstract

**Background:**

Non-neuronal cells, such as microglia and lymphocytes, are thought to be involved in the pathogenesis of amyotrophic lateral sclerosis (ALS). Previous studies have demonstrated neuroprotective effects of lymphocytes at the end stage of ALS, partly through induction of alternatively activated microglia (M2 microglia), which are neuroprotective. In this study, we investigated the role of lymphocytes in the early stage of the disease using an animal model of inherited ALS.

**Methods:**

We established a transgenic mouse line overexpressing the familial ALS-associated G93A-SOD1 mutation (harboring a single amino acid substitution of glycine to alanine at codon 93) with depletion of the Rag2 gene (mSOD1/RAG2-/- mice), an animal model of inherited ALS lacking mature lymphocytes. Body weights, clinical scores and motor performance (hanging wire test) of mSOD1/RAG2-/- mice were compared to those of mutant human SOD1 transgenic mice (mSOD1/RAG2+/+ mice). Activation of glial cells in the spinal cords of these mice was determined immunohistochemically, and the expression of mRNA for various inflammatory and anti-inflammatory molecules was evaluated.

**Results:**

Clinical onset in mSOD1/RAG2-/- mice was significantly delayed, and the number of lectin-positive cells in spinal cord was increased at the early stage of disease when compared to mSOD1/RAG2+/+ mice. Quantitative RT-PCR confirmed that mRNA for Ym1, an M2 microglial-related molecule, was significantly increased in mSOD1/RAG2-/- mouse spinal cords at the early disease stage.

**Conclusions:**

Compared with mSOD1/RAG2+/+ mice, mSOD1/RAG2-/- mice displayed delayed onset and increased M2 microglial activation at the early stage of disease. Thus, lymphocytes at the early pathological phase of ALS display a deleterious effect via inhibition of M2 microglial activation.

## Background

Amyotrophic lateral sclerosis (ALS) is characterized by a progressive degeneration of motor neurons in brain and the spinal cord, resulting in muscle weakness. Patients eventually become paralyzed and approximately 50% die within 3 years of onset of symptoms, usually as the result of respiratory failure [1]. Although the precise mechanisms of ALS remain unclear, approximately 2% of patients with ALS have dominant mutations in the Cu/Zn superoxide dismutase 1 (SOD1) gene [2]. Transgenic mice overexpressing the mutant human SOD1 gene (mSOD1 mice) develop progressive motor neuron degeneration that resembles ALS and therefore these mice serve as an appropriate animal model for the disease [3].

Although ALS is characterized by motor neuron degeneration, activation of microglia and astrocytes and infiltration of T lymphocytes are significant pathological hallmarks in the spinal cord lesions of ALS patients and mSOD1 mice, and a role for these cells in the pathogenesis of ALS has been suggested [4-6]. Recent experiments in mSOD1 mice suggest that neurons do not die alone, but rather that the process is non-cell-autonomous and depends on the active participation of non-neuronal cells, such as microglia, astrocytes, and T cells [7-9].

Microglia, resident immune effector cells in the central nervous system (CNS), display functional plasticity during activation, which involves changes in cell number, morphology, surface receptors, and production of growth factors and cytokines [10]. T-cell-derived cytokines play critical roles in the control of the microglial phenotype. For example, classically activated microglia (M1 microglia) differentiate in response to granulocyte macrophage colony-stimulating factor (GM-CSF) and are primed by interferon gamma (IFN-γ), one of the most important cytokines produced by T helper 1 (Th1) cells, in the presence of lipopolysaccharide (LPS) [10,11]. M1 microglia secrete increased proinflammatory cytokines, superoxide radicals, nitric oxide (·NO), and reduced neurotrophic factors, which promote neuronal death [12]. In contrast, representative T helper 2 (Th2) cytokines, such as interleukin 4 (IL-4) and interleukin 13 (IL-13), can convert microglia, primed by macrophage colony-stimulating factor (M-CSF), to an alternatively activated M2 phenotype [12]. M2 microglia are also characterized by increased expressions of arginase 1 (Arg1), resistin-like alpha (Retnla), and chitinase 3-like 3 (Ym1), which play important roles in tissue repair and remodeling [10]. However, the precise roles of crosstalk between T cells and microglia in the pathology of ALS remain unknown.

In this study, we established mSOD1 mice lacking recombination-activating gene 2 (mSOD1/RAG2-/-), an animal model for inherited ALS that lacks mature lymphocytes, and compared their phenotype and microglial characteristics with that of mutant human SOD1 transgenic mice (mSOD1/RAG2+/+). The clinical onset of mSOD1/RAG2-/- mice was significantly delayed compared to the control group. Consistent with this, increased numbers of activated microglia/macrophages and the expression of Ym1, a molecule with matrix reorganization and wound-healing effects [13,14], were observed at the early stage of the disease in mSOD1/RAG2-/- mice compared to mSOD1/RAG2+/+ mice. These results suggest an important role for lymphocytes interacting with microglia in the early phase of neurodegeneration in ALS.

## Methods

### Animals

All mice were housed in microisolator cages within a modified pathogen-free barrier facility at the Animal Resource Center for Infectious Diseases, Research Institute for Microbial Diseases, Osaka University. All animals had free access to food and water *ad libitum*, and all of the experimental procedures followed our institutional guidelines.

Transgenic mice overexpressing the familial ALS-associated G93A SOD1 mutation (harboring a single amino acid substitution of glycine to alanine at codon 93) (mSOD1 mice) were obtained from The Jackson Laboratory (Bar Harbor, ME) (strain designated: B6SJL-TgN(SOD1-G93A)1Gurd/J) and were backcrossed with C57BL/6 mice for at least 10 generations. Transgenic progeny were identified by a polymerase chain reaction (PCR) of genomic DNA using specific primers for exon 4 of the human SOD1 gene: ex4Pla 5'-CATCAGCCCTAATCCATCTGA-3' and ex4P2a 5'-TGGATCTTAGAATTCGCGAC-3'. PCR conditions included 35 cycles at 94°C for 30 s, 60°C for 30 s and 72°C for 1 min. The PCR products were resolved on a 1.0% agarose/ethidium bromide gel and photographed under UV illumination.

RAG2-/- mice on a C57BL/6 background were obtained from The Jackson Laboratory. RAG2-/- mice were initially bred with mSOD1 mice. The presence or absence of the RAG2 gene was determined by PCR using 250 ng of tail DNA. The following primers were: Rag A, 5'-TAAAAGACCTATTCACAATCAAAAATGTCC-3'; and Rag B, 5'-TCAATCGTGTTGTCCCCTAGAGAGACAAGG-3'. PCR products for the different mice include: a 1100-bp band for homozygote mice, 1100- and 917-bp bands for heterozygote mice, and a 917-bp band for wild-type. PCR was conducted with 35 cycles at 94°C for 30 s, 60°C for 30 s and 72°C for 1 min. The PCR products were resolved by 1.0% agarose/ethidium bromide gel electrophoresis and photographed under UV illumination.

### Symptomatic analysis and hanging-wire test

Symptomatic analysis and weight measurements of male mice were conducted every 3-4 days starting at day 64 (n = 13 for mSOD1/RAG2+/+ mice, n = 11 for mSOD1/RAG2-/- mice). Animals were scored for motor symptoms using a 4-point scoring system as follows: 4 points, normal (no sign of motor dysfunction); 3 points, hind limb tremors were evident when suspended by the tail; 2 points, gait abnormalities were present; 1 point, dragging of at least one hind limb; and 0 points, inability to right self within 30 s. Onset was defined retrospectively as the earliest time when the mice showed symptoms (i.e., score < 4).

For the hanging-wire test, each male mouse was placed on a wire lid of a conventional housing cage. The lid was shaken gently to prompt the mouse to hold onto the grid before the lid was swiftly turned upside down. The latency until the mouse let go with at least both hind limbs was timed. Each mouse was allowed up to three attempts to hold on to the inverted lid for an arbitrary maximum of 90 s and the longest latency was recorded.

### Histochemistry and quantification of microglia and astrocytes

Mice were anesthetized with an overdose of pentobarbital (60 mg/kg i.p.) and transcardially perfused with ice-cold 4% paraformaldehyde. Spinal cords were dissected out, postfixed in the same fixative for 4 h, placed overnight in 30% sucrose, and embedded in O.C.T. Compound (Sakura Finetek, Tokyo, Japan). Sections were frozen in liquid nitrogen and the blocks were stored at -80°C until use. Ten-micrometer-thick transverse sections of spinal cords were prepared using a Leica 3050 S cryostat (Thermo Shandon, Inc., Pittsburgh, PA) and collected onto Superfrost slides (Matsunami Glass Industries, Ltd., Osaka, Japan).

For lectin staining, sections were permeabilized with 0.2% Tween-20 in 0.05 M Tris-buffered saline (TBST, pH 7.2) for 10 min. They were then incubated with FITC-conjugated lectin (Sigma, St. Louis, MO) diluted 1:750 in phosphate-buffered saline (PBS) overnight at 4°C. Sections were washed 3 times in 0.2% TBST for 5 min, and mounted with VECTASHIELD Mounting Medium with DAPI (Vector Laboratories, Burlingame, CA). The fluorescence-labeled sections were examined using a LSM 510 confocal microscope (Zeiss, Thornwood, NY).

For immunohistochemistry, sections were permeabilized with 0.2% TBST for 10 min and pretreated with 10% normal goat serum (Jackson ImmunoResearch Laboratories, West Grove, PA) for 1 h at RT to block non-specific IgG binding. Sections were then incubated with rabbit anti-mouse glial fibrillary acidic protein (GFAP) antibody (Ab) (1:1000) (Dako, Carpinteria, CA) overnight at 4°C. As a negative control, the primary Ab was omitted during the reaction. After rinsing with 0.2% TBST 3 times, sections were incubated with Cy5-conjugated F(ab') 2 fragment donkey anti-rabbit IgG (1:500 in PBS) for 3 h at RT. Sections were then washed 3 times in 0.2% TBST for 5 min and mounted with VECTASHIELD Mounting Medium with DAPI (Vector Laboratories). The fluorescence-labeled sections were examined using an LSM 510 confocal microscope (Zeiss).

For quantification of microglia and astrocytes, we analyzed the density of lectin- or GFAP-positive cells, respectively, in every third sample section of lumbar horizontal spinal cord sections. A total of 25 sections were analyzed and cell densities were counted in 4 randomly selected fields per section (a total of 100 fields).

### Western blots

Mice were anesthetized with an overdose of pentobarbital (60 mg/kg i.p.) and transcardially perfused with ice-cold PBS. Dissected spinal cords were homogenized with protease inhibitors and loaded onto 10% SDS-PAGE gels. Blots were incubated with an antibody against Iba-1 (Wako, Richmond, VA) and visualized by chemiluminescence.

### Quantitative RT-PCR

RNA was isolated from homogenized flash-frozen mice spinal cords using TRIzol (GIBCO/BRL, Grand Island, NY) and purified using RNeasy (QIAGEN, Valencia, CA) according to the manufacturer's recommendations. The concentrations were determined spectrophotometrically. cDNA was generated from 100 ng of each RNA sample digested with RNase-free DNase I (QIAGEN) and SuperScript VILO cDNA Synthesis Kit (Invitrogen, Carlsbad, CA) according to the manufacturer's recommendations. The synthesized cDNA was amplified with SYBR Premix Ex Taq II (Takara Bio Inc., Shiga, Japan) and the primer set, which is listed below, using the StepOnePlus real-time PCR system (Applied BioSystems, Foster City, CA). Primer sets used in this study were: glyceraldehyde-3-phosphate dehydrogenase (GAPDH): 5'-TGTGTCCGTCGTGGATCTGA-3' and 5'-TTGCTGTTGAAGTCGCAGGAG-3'; nitric oxide synthase 2 (NOS2): 5'-GCAGAGATTGGAGGCCTTGTG-3' and 5'-GGGTTGTTGCTGAACTTCCAGTC-3'; IFN-γ: 5'-CGGCACAGTCATTGAAAGCCTA-3' and 5'-GTTGCTGATGGCCTGATTGTC-3'; Ym1: 5'-TTTGATGGCCTCAACCTGGA-3' and 5'-AGTGAGTAGCAGCCTTGGAATGTC-3'; Retnla: 5'-TCAGCAATCCCATGGCGTATAA-3' and 5'-TCATCAGTATTCACTGGGACCATCA-3'; glial cell line derived neurotrophic factor (GDNF): 5'-CCCACGTTTCGCATGGTTC-3' and 5'-TGGGCAGCTGAGGTTGTCAC-3'; solute carrier family 1 (glial high affinity glutamate transporter), member 3 (SLC1A3): 5'-CTTGTCTCATCCATTGGCCTCA-3' and 5'-CCATTCCCAATAAGTGTCCTGTCTG-3'; Mannose receptor, C type 1 (MRC1): 5'-TCGAGACTGCTGCTGAGTCCA-3' and 5'-AGACAGGATTGTCGTTCAACCAAAG-3'; M-CSF: 5'-GAACAGCCTGTCCCATCCATC-3' and 5'-TGAGGCCAGCTCAGTGCAA-3'; GM-CSF: 5'-AAGGGCGCCTTGAACATGAC-3' and 5'-AAATCCGCATAGGTGGTAACTTGTG-3'; interferon gamma receptor 1 (IFN-γR): 5'-TGACGGGAGCACCTGTTACAC-3' and 5'-TTTCGACCGTATGTTTCGTATGTAG-3'; IL-4: 5'-TCTCGAATGTACCAGGAGCCATATC-3' and 5'-AGCACCTTGGAAGCCCTACAGA-3'; interleukin 4 receptor, alpha (IL-4R): 5'-TGAGCCCGTGGTATCTGCAA-3' and 5'-CCAGATCCCTGTGCCTCAAAC-3'; IL-13: 5'-CAATTGCAATGCCATCTACAGGAC-3' and 5'-CGAAACAGTTGCTTTGTGTAGCTGA-3'; interleukin 13 receptor alpha 1 (IL-13R): 5'-CAGTCTTGCAGCATGGGAACA-3' and 5'-TGAGTCCCTAAGGCCTGGAGATTAC-3'. A standard thermal cycling program was used for all PCRs: 95°C for 60 s, 40 cycles of 95°C for 5 s, and 60°C for 30 s. The relative expression levels of each mRNA were calculated using the ΔΔCt method, normalizing to GAPDH and relative to the control samples.

### Statistical Analyses

**Figure 1:**
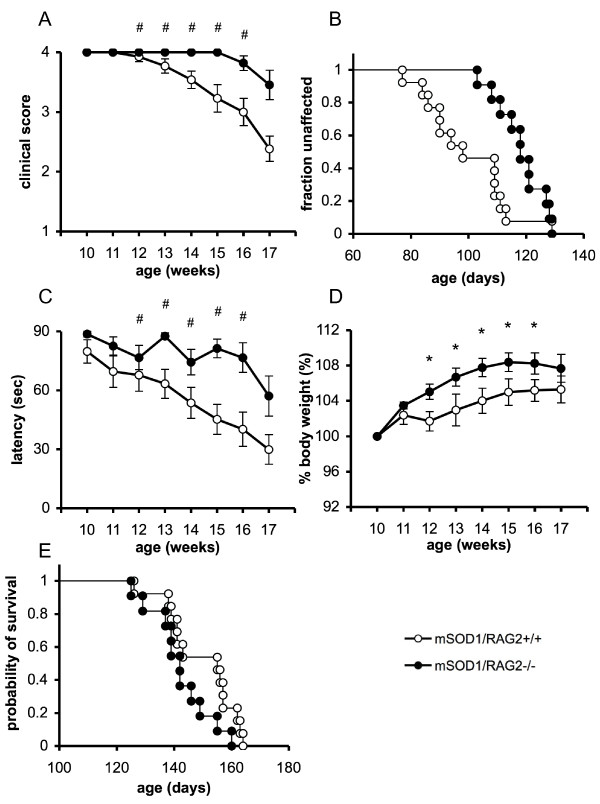
**Delayed clinical onset in male mutant SOD1 transgenic mice deficient in the RAG2 gene**. (A) Disease progression was significantly delayed in mSOD1/RAG2-/- mice (filled circles; n = 11) compared with mSOD1/RAG2+/+ mice (open circles; n = 13). (B) Kaplan Meier curve for symptomatic onset in mSOD1/RAG2+/+ mice (n = 13) and mSOD1/RAG2-/- mice (n = 11). Mice were evaluated for signs of motor deficiency with the following 4-point scoring system: 4 points, normal (no sign of motor dysfunction); 3 points, hind limb tremors were evident when suspended by the tail; 2 points, gait abnormalities were present; 1 point, dragging of at least one hind limb; 0 points, inability to right self within 30 s. We defined the point when the clinical score became 3 as being clinical onset. The mSOD1/RAG2-/- mice exhibited the disease phenotype at a later stage than the mSOD1/RAG2+/+ mice. (C) Time course of motor performance in the hanging-wire test. mSOD1/RAG2-/- mice (n = 11) performed significantly better than mSOD1/RAG2+/+ mice (n = 13). (D) Changes in mean body weight of mSOD1/RAG2-/- mice (n = 11) and mSOD1/RAG2+/+ mice (n = 13). (E) Kaplan-Meier survival curve. Absence of T and B cells did not affect the survival of mSOD1 mice (mSOD1/RAG2+/+ mice; n = 13, mSOD1/RAG2-/- mice; n = 11) (p = 0.13). Data are expressed as the mean ± SEM. Statistical analysis was done using the generalized Wilcoxon test for Kaplan-Meier curves (B,E) and a two-way repeated multivariate ANOVA (A, C, D) during 12 to 16 weeks of age. *:p < 0.05, #:p < 0.005.

Differences in onset and survival (Figure 1B and 1E) were computed using Kaplan-Meier survival statistics (generalized Wilcoxon test). Data in Figure 1A, C and 1D were analyzed using a two-way repeated ANOVA. All other data were analyzed using an unpaired two-tailed Student's t test using Excel software (Microsoft, Dedmond, WA). Data are expressed as mean ± SEM. p < 0.05 was considered statistically significant. **Results**

### Delayed clinical onset in mutant SOD1 transgenic mice deficient in RAG2

To investigate the role of lymphocytes in ALS, mSOD1 mice were crossed with RAG2-/- mice to generate mSOD1/RAG2-/- mice that lack functional T- and B-lymphocytes. Although there were no differences in relative copy number of mSOD1 (data not shown), delayed clinical onset was observed in mSOD1/RAG2-/- mice when compared with mSOD1/RAG2+/+ mice (p = 0.0033) (Figure 1A and 1B) or mSOD1/RAG2+/- mice (mSOD1/RAG2+/- mice: 94 ± 4 days (n = 8); mSOD1/RAG2-/- mice: 118 ± 3 days (n = 11), p = 0.0007, generalized Wilcoxon test). Next, neuromuscular dysfunction in mSOD1 mice was evaluated with the hanging-wire test twice a week. Consistent with the clinical onset and scores, genetic depletion of lymphocytes significantly improved motor function of mSOD1/RAG2-/- mice during the early stage (p = 0.0043) (Figure 1C). As neurodegeneration in mSOD1 mice is accompanied by loss of body weight, we also measured the body weight of these mice. We found that mSOD1/RAG2-/- mice had increased weight gain compared to mSOD1/RAG2+/+ mice, suggesting alleviated neurodegeneration in mSOD1/RAG2-/- mice (p = 0.0457) (Figure 1D). mSOD1/RAG2+/+ mice had normal numbers of white blood cells and normal ratios of T and B lymphocytes (white blood cell count: mSOD1tg 8067 ± 1324/ul, control 10867 ± 1629/ul, percentage of CD3^+ ^cells: mSOD1tg 32.00 ± 1.21%, control 31.18 ± 1.23%, percentage of B220^+ ^cells: mSOD1tg 55.97 ± 2.19%, control 57.85 ± 1.52%, n = 4 for each group). Although the absence of mature lymphocytes delayed onset of the disease, it did not affect the survival of mSOD1 mice (Figure 1E) (p = 0.13). Furthermore, survival times for mSOD1/RAG2+/- mice were comparable to those of mSOD1/RAG2-/- mice (average survival time; mSOD1/RAG2+/- mice: 142 ± 3 days (n = 8); mSOD1/RAG2-/- mice: 142 ± 3 days (n = 11), p = 0.74, generalized Wilcoxon test). Collectively, these results suggest that lymphocytes may contribute to acceleration of neurodegeneration in mSOD1 mice at the early stage.

### Increased microglial activation at the early stage in mSOD1 mice without mature lymphocytes

**Figure 2:**
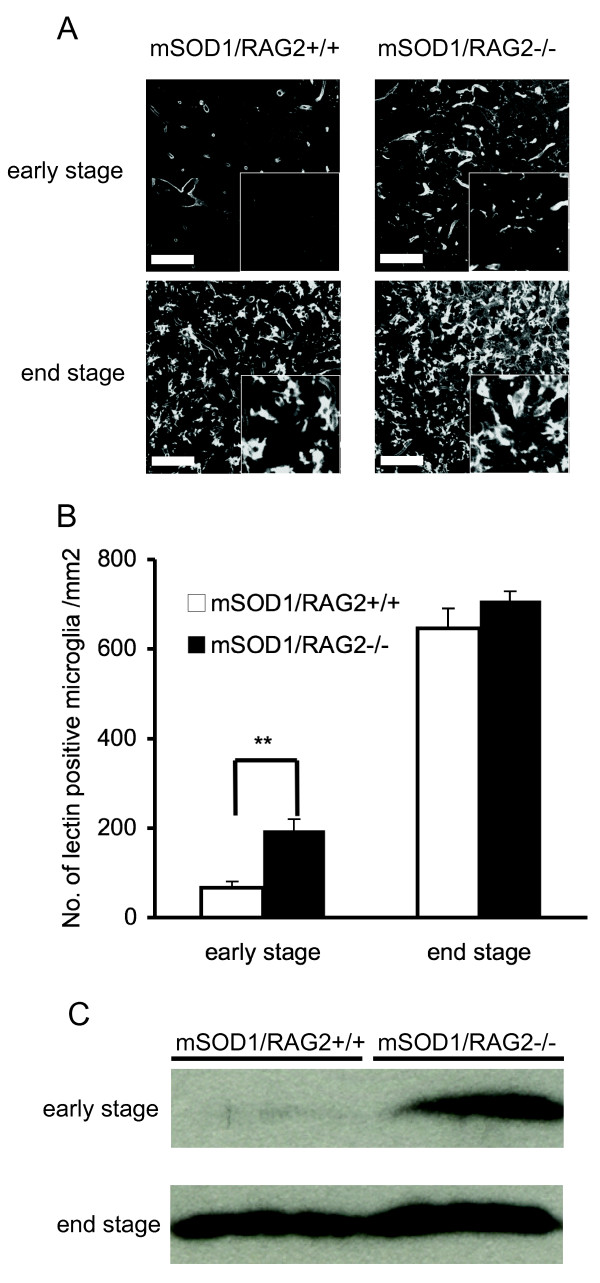
**Significantly increased lectin-positive cells at the early stage of disease**. (A) Lumbar sections of spinal cord from mSOD1/RAG2-/- and mSOD1/RAG2+/+ mice at early (70-90 days of age) and end (130-150 days of age) stage of disease were stained for lectin (microglia/macrophage). Scale bar = 50 μm. (B) Significantly increased numbers of lectin-positive microglia/macrophages are observed at the early stage of the disease in spinal cord sections of mSOD1/RAG2-/- mice compared with mSOD1/RAG2+/+ mice. There was no significant difference in the number of lectin-positive cells at the end stage of disease. Significance was evaluated by using a generalized Wilcoxon test. **: p < 0.01. (C) Western blot analysis of Iba-1 shows markedly increased expression in lumbar spinal cord of mSOD1/RAG2-/- mice compared with mSOD1/RAG2+/+ mice at early stage. Data presented in A-C is representative of three animals.

Neurodegeneration in ALS is accompanied by microglial-mediated neuroinflammation, which greatly affects disease progression [12]. Persistent activation of microglia is achieved by the sustained production of various cytokines induced by T lymphocytes [10]. To assess whether the absence of lymphocytes affects activation of microglia/macrophages in mSOD1 mice, spinal cord sections were stained with lectin. Lumbar spinal cord sections from early stage (70-90 days of age) mSOD1/RAG2-/- mice showed significantly increased numbers of lectin-positive microglia/macrophages compared with mSOD1/RAG2+/+ mice. However, there was no significant difference in the number of these cells between mSOD1/RAG2-/- mice and mSOD1/RAG2+/+ mice at the end stage of disease (130-150 days of age) (Figure 2A and 2B). Consistent with these results, western blot analysis showed an intense band for Iba-1 at the early stage of disease in spinal cord of mSOD1/RAG2-/- mice (Figure 2C). However, this striking difference disappeared at the end stage.

**Figure 3:**
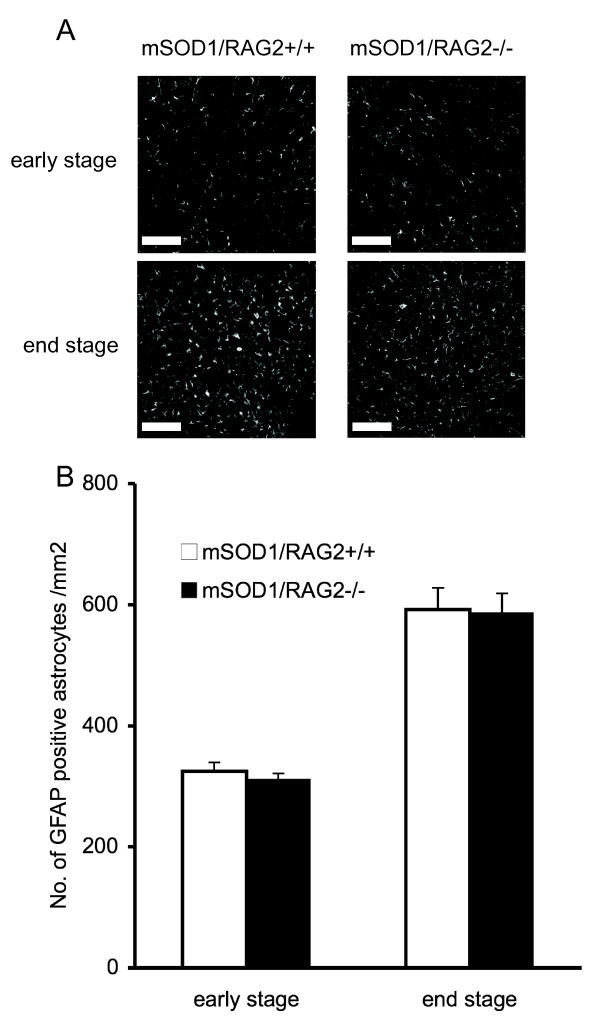
**The number of GFAP-positive cells in spinal cord does not differ between mSOD1/RAG2+/+ and mSOD1/RAG2-/- mice**. (A) Lumbar sections of spinal cord from mSOD1/RAG2-/- mice and control mice stained with anti-GFAP antibody. Scale bar = 50 μm. (B) The number of GFAP-positive astrocytes in lumbar cord of mSOD1/RAG2-/- mice does not significantly differ from that of control mSOD1 mice at early (70-90 days of age) and end (130-150 days of age) stages. Data presented in A-B is representative of three animals.

Astrocytes are the most abundant non-neuronal cells in the CNS and their important roles in neurodegenerative disease have been suggested [8,9]. To assess the relevance of astrocytes, GFAP-immunoreactive cells were evaluated in lumbar spinal cords. At both early and end stages, there were no differences in intensity of GFAP immunoreactivity (IR) between mSOD1/RAG2-/- mice and control mice (Figure 3A). Also, both groups had comparable numbers of GFAP positive cells (Figure 3B). Collectively, these results suggest that mature lymphocytes may play a role in the inhibition of microglial activation but not of astrocyte activation.

### Increased mRNA expression of Ym1, a neuroprotective factor, in mSOD1/RAG2-/- mice at the early stage

**Figure 4:**
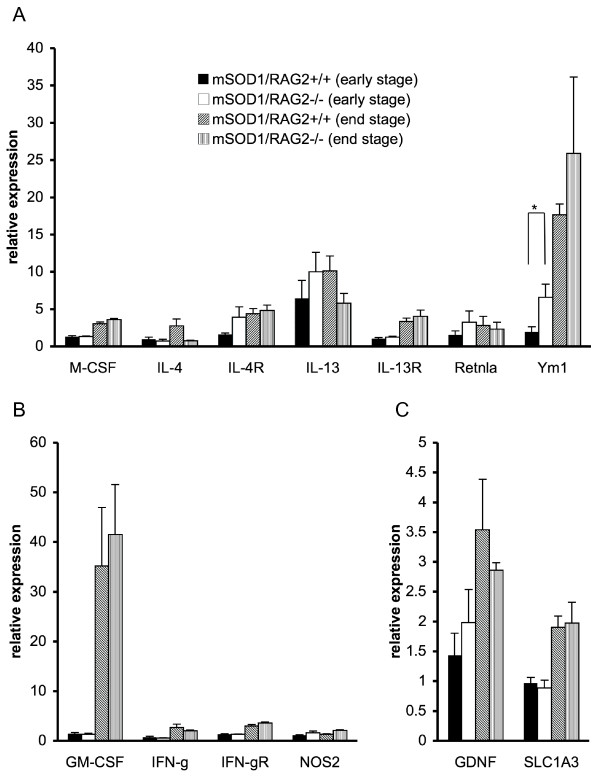
**Expression of Ym1 mRNA is significantly increased in the spinal cord of mSOD1/RAG2-/- mice**. Quantitative RT-PCR analyses of spinal cord of mSOD1/RAG2-/- mice and mSOD1/RAG2+/+ mice at early (70-90 days of age) and end (130-150 days of age) stages were performed (n = 3-4 in each group). (A) Relative mRNA expression of M-CSF, IL-4, IL-4R, IL-13, IL-13R, Retnla, and Ym1, which are related to M2 macrophages, is shown. Ym1 expression levels are significantly increased in mSOD1-RAG2-/- mice at the early stage of disease (p = 0.039). (B) Relative mRNA expression of GM-CSF, IFN-γ, IFN-γ R, and NOS2, which are related to M1 macrophages, is shown. M1 macrophage-related molecules were comparable between mSOD1/RAG2-/- mice and control mice. (C) Relative mRNA expression of GDNF and SLC1A3 is shown. Expression levels of GDNF and SLC1A3 did not differ between mSOD1/RAG2-/- mice and control mice at early and end stages. Significance was evaluated using the student's t test. *: p < 0.05.

Activation of microglia in the presence of GM-CSF and IFN-γ results in a classically activated phenotype (M1 microglia) characterized by the production of ·NO [11]. The primary function of M1 microglia is thought to be activation of inflammation and tissue injury, whereas microglia activated alternatively (M2 microglia) by M-CSF, IL-4 and IL-13 express mannose receptor, MHC class II and Ym1, which leads to resolution of inflammation and promotion of wound healing [10,11]. To determine if delayed onset in mSOD1/RAG2-/- mice is attributable to increased numbers of M2 microglia, we evaluated the mRNA expression of M2 microglial target genes in spinal cord of mSOD1/RAG2-/- mice and control mice. Quantitative RT-PCR demonstrated that mRNA expression of Ym1, which is induced by activated M2 microglia/macrophages, was significantly elevated in mSOD1/RAG2-/- mice compared with mSOD1/RAG2+/+ mice at early stage but not at end stage disease. The expression of IL-4R and IL-13 mRNA tended to be elevated in the mSOD1/RAG2-/- mice at the early stage, although this change was not significant (Figure 4A). In contrast, levels of M1 microglia-related molecules such as GM-CSF, IFN-γ, IFN-γ R and NOS2 were similar between mSOD1/RAG2-/- mice and control mice both at early and end stages (Figure 4B). It is worthy to note that considerable expression of GM-CSF, which can induce M1 microglia/macrophages, was observed at the end stage of disease in both groups. There were no significant differences in the expression of mRNA for neurotrophic factors such as GDNF and SLC1A3 between the two groups (Figure 4C). These results suggest that M2 microglia are more dominant than M1 microglia in the spinal cord of mSOD1/RAG2-/- mice at the early stage.

## Discussion

In this study, we demonstrate delayed clinical onset in mSOD1/RAG2-/- mice compared to mSOD1/RAG2+/+ mice, with increased number of activated microglia in the spinal cord. Microgliosis in mSOD1/RAG2-/- mice was accompanied by increased Ym1 mRNA expression, one of the hallmarks of M2 microglia/macrophages. However, the differences in weakness, microgliosis, and Ym1 expression between SOD1/RAG2-/- and mSOD1/RAG2+/+ mice disappeared in the later stage of disease. This suggests that this affect on M2 microglia/macrophages, as the result of lymphocyte depletion, may be associated with delayed clinical onset in mSOD1/RAG2-/- mice. As a previous study has shown that B lymphocytes do not have a major role in neurodegeneration in G93A-SOD1 mice [15], T lymphocytes rather than B lymphocytes are thought to be responsible for the increased M2 activation of microglia. Although few T lymphocytes have been shown to infiltrate spinal cord in mSOD1 mice in the early stage, T cells in the meninges may have some effects on neurons or glial cells without entering the parenchyma [16-18]. Increased GM-CSF at the late stage of disease, as shown in Figure 4B, might counteract the neuroprotective effect of M2 microglia observed at the early phase.

In contrast to our finding of a deleterious effect of lymphocytes in ALS, previous studies with mSOD1/RAG2-/- mice have indicated a protective rather than deleterious role of lymphocytes in mSOD1 mice [6,16,19]. A possible explanation for these discrepancies is differences in transgene copy number in the mice used by us and other research groups. The mutant G93A transgene is reported to undergo a background level of copy loss due to meiotic rearrangement of the transgene array [20,21]. Since mSOD1 has been suggested to activate innate immunity downstream of the toll-like receptor (TLR) 2, TLR4 and CD14 [22], a difference in transgene copy number may cause different levels of microglial activation. Another possibility is environmental factors. Mice maintained under germ-free conditions develop significantly attenuated symptoms of experimental autoimmune encephalomyelitis (EAE) compared with conventionally colonized mice, and microbiota are reported to have an effect on the T-helper/Treg axis outside of the gut [23]. Thus, different microbiota of mice maintained in different facilities could change the characteristics of lymphocytes and other immune cells in the CNS, thus leading to the discrepancy between our study and previous reports.

Piecing together our results and previous studies suggests that lymphocytes might have biphasic effects on neurons in this animal model of ALS. That is, lymphocytes may have a deleterious effect in the early phase of neurodegeneration by suppressing M2 microglia, whereas they may exert neuroprotective effects in the later stage of the disease, thus leading to the shortened survival of mSOD1/RAG2-/- mice reported in a previous study [6]. Transgene copy number of our mice and the SPF condition of our facilities might be responsible for the apparent difference seen in the early stage of neurodegeneration between the two groups, while those of other facilities appear to affect the end stage.

The present findings suggest that mature lymphocytes act to produce a deterioration of the ALS phenotype in mSOD1 mice at the early stage of the disease by modulating the trophic balance of microglia. If the course of ALS is so modulated, then disease progression could be delayed by expanding populations of M2 microglia/macrophages, thus producing neuroprotective effects.

## Conclusions

This study reports delayed onset of ALS phenotype in mSOD1/RAG2-/- mice compared to controls. We observed an increased number of lectin-positive microglia/macrophages at the early stage of disease in mSOD1/RAG2-/- mice compared with mSOD1/RAG2+/+ mice. In addition, quantitative RT-PCR revealed increased expression of mRNA for M2-microglial-related genes in mSOD1/RAG2-/- mice. In conclusion, these results indicate that peripheral lymphocytes act to produce a deterioration of the ALS phenotype in mSOD1 transgenic mice at the early stage of disease by inhibiting M2 microglia/macrophage skewing.

## Competing interests

The authors declare that they have no competing interests.

## Authors' contributions

ST designed the experiments, analyzed the data, and wrote the manuscript.

TO, TY and YN participated in the design of the study and helped draft the manuscript. TS contributed analysis of data as well as provided editing of the manuscript. HK and SS conceived of the study, and participated in its design and conduct and helped draft the manuscript. All authors have read and approved the final version of the manuscript.
